# Chronic kidney disease at presentation is not an independent risk factor for AIDS-defining events or death in HIV-infected persons 

**DOI:** 10.5414/CN107390

**Published:** 2012-12-27

**Authors:** Tahira P. Alves, Pingsheng Wu, T. Alp Ikizler, Timothy R. Sterling, Samuel E. Stinnette, Peter F. Rebeiro, Suvro Ghosh, Todd Hulgan

**Affiliations:** 1 University of Texas Health Science Center at San Antonio, Department of Medicine, Division of Nephrology, San Antonio, TX,; 2 Department of Biostatistics,; 3 Department of Medicine, Division of Nephrology and; 4 Department of Medicine, Division of Infectious Diseases, Vanderbilt University Medical Center, Nashville, TN, USA

**Keywords:** HIV, CKD, AIDS defining event (ADE), mortality

## Abstract

Studies have documented an association between chronic kidney disease (CKD) and increased risk of end-stage renal disease (ESRD), death and comorbidities, including cardiovascular disease and metabolic syndrome, in the general population. However, there is little data on the relationship between CKD and ADE (AIDS defining event), and to our knowledge, no studies have analyzed death as a competing risk for ADE among HIV-infected persons. An observational cohort study was performed to determine the incidence and risks for developing an ADE or death among HIV-infected persons with and without CKD from 1998 – 2005. CKD was defined as an estimated glomerular filtration rate (eGFR) less than 60 ml/min/1.73 m^2^ using the CKD-Epidemiology Collaboration (CKD-EPI) equation. Log rank test and Cox regression which determined time to development of ADE and/or death as combined and separate outcomes, and competing risk models for ADE versus mortality, were performed. Among the 2,127 persons that contributed to the 5,824 person years of follow-up: 22% were female, 34% African-American, 38% on HAART, and 3% had CKD at baseline. ADE occurred in 227 (11%) persons and there were 80 (4%) deaths. CKD was not significantly associated with ADE/death (HR 1.3, 95% CIs: 0.5, 3.2), ADE (HR 1.0, 95% CIs: 0.4, 3.1), or death (HR 1.6, 95% CIs: 0.4, 3.1). Competing risk analyses confirmed no statistically significant associations between CKD and these outcomes. CKD was uncommon in HIV-infected persons presenting for care in this racially diverse cohort, and was not independently associated with risk of developing an ADE or dying during follow-up.

## Introduction 

Chronic kidney disease (CKD), a well-recognized national health epidemic, affects ~ 26 million people in the United States [1]. Studies have documented the association between CKD and an increased risk of end-stage renal disease, death and comorbidities, including cardiovascular disease and metabolic syndrome, in the general population [2, 3, 4, 5]. These associations have also been observed in HIV-infected individuals, especially among black persons [6, 7, 8, 9]. However, the effect of CKD on the development of AIDS defining events (ADE) in the HIV-infected population is less well established. Some well recognized examples of ADE include Pneumocystis jiroveci pneumonia, cytomegalovirus, and cryptosporidiosis [10]. Earlier studies have documented an increased risk of an ADE and mortality among HIV-infected women with increased creatinine and proteinuria during the early era of highly active antiretroviral therapy (HAART) [11]. Studies from the same cohort have recently confirmed a significant association between decreased level of kidney function at HAART initiation and mortality in subjects during the later HAART period [12]. 

To our knowledge no studies have analyzed death as a possible competing risk for ADE among a cohort of HIV-infected men and women. The analysis of death as a potential competing risk for ADE is important because it establishes the likelihood of an individual that was initially categorized as dying and not developing an ADE would develop an ADE if the subject had not died during the inclusion period of the analysis. This form of analysis potentially decreases the chances of underestimating the risk of ADE due to premature mortality in the event that death is a competing risk for ADE. Therefore, the aim of the current study was to determine the effect of CKD on the occurrence of ADE and mortality among HIV-infected individuals receiving care in middle Tennessee (TN) during the HAART era. It was hypothesized that HIV-infected individuals with CKD at presentation for care – a potentially modifiable risk factor which may facilitate early intervention – and those that developed CKD during the study follow-up period, would have a greater risk for ADE and death, as compared to HIV-infected individuals with no CKD. 

## Materials and methods 

### Study population and study follow-up

The Comprehensive Care Center (CCC) is a clinic in Nashville, Tennessee, has treated HIV-infected persons in Middle Tennessee since 1994. Entry into this retrospective cohort study was the date of first serum creatinine that was followed by a second serum creatinine measurement collected ≥ 90 but < 365 days from the first measurement, within the study period (January 1, 1998 – December 31, 2005). Individuals were censored at death or occurrence of ADE, whichever occurred first during the study period. An individual was defined as lost to follow-up if they had more than a 1-year gap between the last provider visits or laboratory values by the end of the study period or the date of their death. Individuals for whom eGFR could not be calculated or who did not have at least two serum creatinine measurements at least 90 – 365 days apart within the study period were excluded. In addition, individuals with ADE at baseline were also excluded from the study. 

### Data sources, CKD diagnoses, and outcomes

Data obtained from the CCC clinical electronic medical records (EMR) dated January 1, 1998 through December 31, 2005 were used for all analyses and to calculate eGFR using the CKD-Epidemiology Collaboration (CKD-EPI) equation as the primary outcome [13]. Clinical data were entered into the EMR at the time of the patient encounter, by automated data upload from reference laboratory results, or by clinic personnel. Antiretroviral therapy was validated by systematic chart review. However, adherence to HAART was not assessed in this study. The Vanderbilt University Institutional Review Board approved this study with waiver of informed consent. All analyses used aggregate, de-identified data. 

Subjects were classified as having CKD at baseline with eGFR ≤ 60 ml/min/1.73 m^2^ that were based on two measurements within the inclusion period. Subjects who did not have CKD at baseline were considered as having CKD at the time when their eGFR progressed to ≤ 60 ml/min/1.73 m^2^ and at least 50% decrease of baseline eGFR during the study period. The outcomes analyzed included ADE, the combined outcome ADE and death, and death alone. ADEs were identified on the basis of 1993 Centers for Disease Control and Prevention (CDC) classification criteria, excluding diagnoses based on CD4 cell counts < 200 cells/mm^3 ^[10]. Hypertension, Hepatitis C, anemia, diabetes mellitus, and cardiovascular disease diagnoses were based on International Classification of Diseases Ninth Revision (ICD-9) Code (Supplemental Table 1). 

### Statistical analyses

Descriptive statistics were expressed as frequencies and proportions for categorical variables and as means and standard deviation (SD), or median and interquartile ranges (IQR) for continuous variables depending on their distribution. Comparisons of renal and HIV parameters at baseline between groups with and without CKD were performed by using the χ^2^-test for categorical variables, and by using the Mann-Whitney U-test for continuous variables. Incidence rates per 1,000 patient years were calculated. 

Cox proportional hazard regression models were used to compute hazard ratios (HRs) of ADE, death, and combined outcome of ADE and death from the time of enrollment for participants with and without CKD [14]. Multivariable Cox regression analyses adjusted for sex, race, baseline age, level of kidney function, anemia status, cardiovascular disease (yes/no), absolute CD4+ lymphocyte count (CD4), HIV-1 RNA viral load (VL), history of angiotensin converting enzyme inhibitor (ACEI) or angiotensin receptor blocker (ARB) (yes/no), HAART status (yes/no), hypertension status (yes/no), chronic hepatitis C status (yes/no), diabetes mellitus status (yes/no), and HIV risk group (intravenous drug use versus no other risk factors) were performed. Covariates were chosen a priori based on clinical relevance. Assumptions of proportional hazards for the final models were evaluated and met [14]. 

Competing risk analyses were performed to consider the different causes contributing to ADE and death among participants with and without CKD. To assess the predictive value of CKD for ADE and death, we used the proportional subdistribution hazard model [15]. The association of CKD with ADE and death was further evaluated with Cox proportional hazards regression using CKD as a time-varying exposure adjusting for the previously listed covariates. All data analyses were performed with R-software version 2.11.1 (2009) [16]. A significance level of 0.05 was used for all statistical inferences. 

## Results 

### Baseline individual characteristics

Among 2,127 individuals included in the analysis, 66 subjects were classified as having CKD at baseline. Among those who were not classified as CKD at baseline, 33 subjects developed CKD during the follow-up period. Our median follow-up time for the study cohort was 2.1 years (IQR: 1.0, 3.7 y). Table 1 depicts the baseline characteristics of the cohort by the presence or absence of baseline CKD. Of the 2,127 individuals, 1,655 (78%) were male, 713 (34%) were self-identified AAs and 1,414 (66%) non-AAs. There were no significant differences between the prevalence of CKD between men and women (p = 0.11). However, a larger percentage of individuals with CKD were African-American compared to non-African-Americans (56% vs. 44%, respectively, p < 0.01). Although individuals with CKD were more likely to have a decreased absolute CD4 count (cells/mm^3^) (p < 0.01), there was no significant difference in HIV-1 RNA VL (copies/ml) between individuals with CKD compared to those without CKD at baseline (p = 0.86). Finally, individuals with CKD at baseline had a higher prevalence of intravenous drug use, cardiovascular disease, diabetes mellitus, Hepatitis C, and anemia (p < 0.05). 

### Long-term outcomes among the entire cohort

A composite outcome was defined as first ADE or death. The incidence rate ratios and hazard rates are reported for all subgroup analyses in Table 2. There were 277 composite events among the 2,127 individuals during the follow-up period, 262 composite events (216 ADE and 73 death) in the 1,850 subjects with no CKD at baseline group and 15 composite events (11 ADE and 7 death) in the 66 subjects with CKD at baseline group (Table 2). A total of 30 individuals had ADE and later died (27 in no CKD at baseline group and 3 in the CKD at baseline group). Although the risk for the composite event was 30% higher for individuals with CKD at baseline compared to individuals with no CKD at baseline (adjusted HR 1.3, 95% CIs 0.5, 3.2), the increased risk was not statistically significant. ADE and death were further analyzed separately to assess whether there was a statistically significant difference in risk for each outcome. However, after adjusting for covariates, individuals with CKD at baseline did not have a significantly increased risk of ADE (adjusted HR 1.0, 95% CIs 0.4, 3.1) or death (adjusted HR 1.6, 95% CIs 0.4, 7.3). In addition, there was no significant difference between different ADE illnesses when stratified by the presence or the absence of CKD (Supplemental Table 1). 

### Competing risk analysis

**Figure 1.**
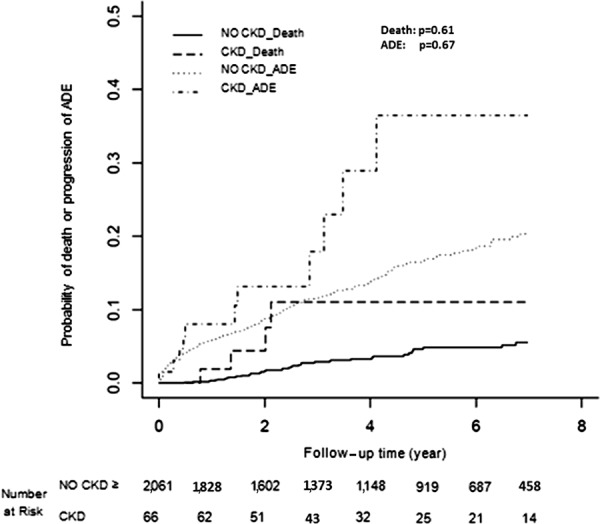
Cumulative incidence of ADE and death among HIV+ comprehensive care center (CCC) Individuals based on chronic kidney disease (CKD) exposure with ADE and death were considered as competing risks. Model was adjusted for the following baseline covariates: age, absolute CD4 count, HIV-1 RNA, race, gender, hypertension, anemia, HAART use, hepatitis C, OI/ADE, IDU HIV risk, ACEI/ARB use, and anemia.

Competing risk models were performed to determine whether there was a differential risk of ADE or death between individuals with and without CKD at baseline assuming that death prevents the observation of ADE (Figure 1). Death was not a significant competing risk for ADE when comparing individuals with and without CKD within our analyses. In summary, the risk of death did not mask the risk of ADE among individuals with or without CKD, even after controlling for covariates, in the competing risk analysis. 

### Time varying cox regression analysis

Time varying Cox regression analyses of the effect of CKD on the development of ADE and death were performed to assess the effect of the development of CKD after baseline on the risk of ADE and death during the study period. Subjects without CKD at baseline were reclassified into the CKD group at the time when they developed CKD. In total, 33 subjects (1.6% of those without baseline CKD) developed CKD during follow-up (IR 5.8 per 1,000 person years). These results were consistent with those only considering CKD status at baseline (Table 3). Progression to CKD during the follow-up period did not increase the risk of ADE or death. 

## Discussion 

The current study was undertaken to explore the effect of CKD on the risk of ADE and mortality among HIV-infected men and women. Moderate to severe CKD was present in 3% of HIV-infected persons presenting for care in this racially diverse cohort. HIV-infected individuals with CKD at baseline did not demonstrate a significantly increased risk of developing an ADE. Individuals with CKD at baseline had a lower absolute CD4 count than individuals without CKD. This finding may have potentially signified more advanced HIV infection in individuals with CKD, and consequently, explained a higher risk of death among individuals with CKD. However, the increased death rate among individuals with CKD was attenuated and no longer statistically significant in multivariable analyses. Individuals who did not have CKD at baseline but developed CKD during the course of the observation period also did not demonstrate a significantly increased risk for the development of ADE or death compared to individuals who remained without CKD during the study period in time varying Cox Regression analyses. Low absolute CD4 count and increased viral load were independently associated with increased risk for all outcomes, but these trends were also attenuated in multivariable analyses. A competing risk analysis was performed to determine whether the risk of developing ADE among individuals with CKD was masked by an increased incidence of death in the study. There was no significant risk for death versus ADE in the competing risk analysis after controlling for covariates among individuals with CKD when compared to those without CKD. 

An earlier study involving HIV-infected women cared for during the early HAART era demonstrated that subjects with an elevated creatinine and proteinuria were at an increased risk of death and AIDS defining illness, a finding that was not confirmed in our study. The association between CKD and increased risk of death within this same cohort of HIV-infected women was further corroborated in a follow-up study during the later HAART era [9, 10]. Of note, the study conducted in the early HAART era used a decreasing inverse creatinine level as a predictor of loss of kidney function. A follow-up of the same study conducted in the late HAART era used an eGFR ≤ 60ml/min/1.73 m^2^ based on the MDRD), as a predictor of chronic kidney disease. Proteinuria was defined by urine dipstick measurement [10]. 

There are several possible reasons why our current study might have not demonstrated a consistent finding with the earlier reports. While our study included a more stringent criterion for classification of CKD by using the CKD-EPI, it is limited by the lack of information on proteinuria [11]. It is possible that early screening for proteinuria to evaluate the presence or absence of CKD in HIV-infected patients may be just as crucial as measurement of eGFR because elevations in proteinuria may manifest before a decline in eGFR; a finding which has been well established in individuals with early diabetic nephropathy [17]. As a result, the lack of significance in risk of adverse outcomes in HIV-infected patients with CKD in the current study may suggest that proteinuria may be a better predictor of adverse outcomes, especially in populations that receive late or no treatment with HAART. The role of early proteinuria and increased risk of co-morbidities including cardiovascular disease, diabetes, and progression to ESRD has been well documented in the general population [18, 19, 20, 21, 22, 23]. More studies that are specifically designed to evaluate the role of proteinuria and risk of ADE and death in vulnerable HIV-infected populations are needed. 

Another important observation in this study is that African-American ethnicity did not modify the risk for ADE or death when compared to non-African-American HIV-infected individuals without CKD in the multivariable analysis. We previously demonstrated that HIV-infected African-Americans and non-African-Americans with no CKD at baseline (e.g. eGFR ≥ 60 ml/min/1.73 m^2^) had a similar risk for rapid decline in eGFR (e.g., greater than 50% decrease in baseline eGFR) [24]. In contrast, HIV-infected African-Americans with baseline CKD (e.g., eGFR < 60 ml/min/1.73 m^2^) tended to have a higher incidence of rapid eGFR decline compared to HIV-infected non-AAs, but these risks did not reach statistical significance. Our current findings pertaining to the overall risk of death and CKD for the entire cohort are consistent with our prior study, which demonstrated that there was no overall increased risk for death for individuals stratified by the presence or absence of CKD, and is extended to include ADE. The small sample size within sub-group analyses might be an explanation for these observations. 

Our study has several limitations and strengths. First, we cannot rule out the possibility of residual confounding by unmeasured factors in this observational study including duration of treatment with HAART therapy, adherence to medications, especially HAART, proteinuria, and important socioeconomic factors including duration of access to medical care and insurance status. In addition, the etiology of kidney disease and proteinuria data was not available and there was a significant lack of power which may have limited our ability to observe a significant association with CKD exposure on ADE and death due to the limited number of individuals with CKD during the study period. Strengths of the current study include the provision of further insight into the effect of CKD on the occurrence of ADE within a well-defined cohort of HIV individuals during the HAART era. To our knowledge, this is one of the few studies that use the CKD-EPI equation to evaluate eGFR within a HIV-infected population. The CKD-EPI equation, as opposed to earlier equations used to estimate eGFR was used in the current study because of the large number of HIV-infected individuals with an eGFR measurement equal to or greater than 60 ml/min/1.73 m^2^. However, more studies are needed that validate the best method to assess GFR among HIV-infected individuals. 

In conclusion, our results demonstrated that HIV-infected patients with CKD did not have significantly increased risk of ADE or death compared to those without CKD among individuals within a large and diverse cohort. This observation was further corroborated in the time varying Cox analysis, which takes into account risk of ADE and death for HIV infection of individuals who did not have CKD at baseline, but develop CKD during follow-up time. Our findings also did not demonstrate that the risk of ADE was masked by risk of death in the competing risk analysis. The specific reasons for the differences observed in this study compared to prior studies which observed an increased risk of ADE and death in HIV-infected individuals with CKD are unclear, but are likely confounded by differences in defining eGFR, differences in method for assessing CKD, and lack of data pertaining to level of proteinuria in our current study. 

## Acknowledgments 

This work was supported by the following research grants: 5 T32 DK007569-17 and K24 DK62849 from the National Institute of Diabetes, Digestive and Kidney Diseases (TAI), National Kidney Foundation Research Fellowship Award (TPA), National Center on Minority Health and Health Disparities/National Institutes of Health Loan Repayment Award (TPA), Vanderbilt University Clinical Translational Science Award 1UL-1RR024975 (TPA), Vanderbilt-Meharry Center for AIDS Research (National Institutes of Health P30 AI054999 – TRS, SES, PFR), K23 AT002508 from the National Center for Complementary and Alternative Medicine (TH), The Tennessee Valley VA Clinical Research Center of Excellence (TH), and the National Center for Research Resources and National Institutes of Allergy and Infectious Diseases K24 A1065298 (TRS, SES). 

**Supplemental Table 1.** ICD-9 Codes.

The following diagnosed conditions defined as an established diagnosis by the International Classification of Diseases Ninth (ICD-9) Revision diagnostic codes: coronary artery disease/cardiovascular disease (Keywords: established previous diagnosis of cardiovascular disease, myocardial ischemia, infarction, congestive heart failure, systolic dysfunction, and diastolic dysfunction; ICD-9 Code: 402, 404, 410, 414, 428, 429, 440, 794.30, and 794.31), hypertension (Keywords: established diagnosis of hypertension, renovascular hypertension, benign, essential or malignant hypertension; ICD 9 Codes: 401.0, 401.1, 403.01, 403.91, 404.13, and 405.01), diabetes (Keywords: diabetes with renal manifestations, diabetic nephropathy, and Type II diabetes; ICD 9 Codes: 250.0, 250.60, 250.40, and 250.70), anemia (Keywords: iron deficiency anemia, pernicious anemia, anemia of chronic disorder, anemia of infection, macrocytic anemia, megaloblastic anemia, glucose-6-phosphate dehydrogenase deficiency anemia, sickle cell anemia, anemia due to decreased red cell production, anemia of chronic renal failure, anemia not otherwise specified, anemia unspecified, and anemia due to unknown mechanism not otherwise specified; ICD 9 Codes: 280, 280.9, 280.1, 281.9, 282.2, 282.6, 285.2, 285.8, 285.9), CKD/ESRD (Keywords: chronic kidney disease, end-stage renal disease (ESRD), HIV associated nephropathy (HIVAN), focal segmental glomerulosclerosis (FSGS), proteinuria, diabetic nephropathy, diabetes with renal manifestations, and chronic interstitial nephritis; ICD 9 Codes: 585.1, 585.2, 585.3, 585.4, 585.5, 585.6, V45.1, 585.9, 593.89, 791.0, and 582.89)

**Table 1.** Baseline characteristics of HIV positive individuals cared for at the comprehensive care center (CCC), 1998 – 2005.

Category	N	No CKD n or %	CKD n or %	p value
	2,127	2,061 (97%)	66 (3%)	
Gender	2,127			
Male	1,655	1,609 (78%)	46 (70%)	0.11^1^
Female	472	452 (22%)	20 (30%)	
Race	2,127			
Non AA	1,414	1,385 (67%)	29 (44%)	< 0.01^1^
AA	713	676 (33%)	37 (56%)	
Median age (y)^a^	2,127	38	48	< 0.01^2^
Median creatinine (mg/dl)	2,127	0.9	1.6	< 0.01^2^
Median weight (kg)	2,127	77	73	0.11^2^
Median BMI (kg/m^2^)	2,112	25	25	0.11^2^
Median calculated eGFR^b^				
CKD-EPI	2,127	104	49	< 0.01^2^
MDRD	2,127	100	48	< 0.01^2^
HIV Risk Group: IDU	2,127	303 (15%)	12 (18%)	< 0.01^1^
Median absolute CD4 count (cells/mm^3^)^d^	2,064	360	256	< 0.01^2^
Median HIV-1 RNA VL (copies/ml)^e^	2,013	7,080	6,174	0.86^2^
Median serum albumin (g/dl)	2,123	4.3	3.8	< 0.01^1^
HAART at baseline	2,127	784 (38%)	26 (39%)	0.82^1^
HAART use before baseline	1,317	184 (14%)	2 (5%)	0.09^1^
ACEI/ARB at baseline	2,127	88 (4%)	12 (18%)	< 0.01^1^
Tenofovir at baseline	2,127	78 (4%)	5 (8%)	0.12^1^
Comorbid conditions^f^				
Cardiovascular disease	2,127	87 (4%)	14 (21%)	0.03^1^
Diabetes mellitus	2,127	146 (7%)	10 (15%)	0.01^1^
Hypertension	2,127	486 (26%)	71 (31%)	0.07^1^
Hepatitis C	2,127	516 (25%)	41 (62%)	< 0.01^1^
Anemia	2,127	243 (12%)	24 (36%)	< 0.01^1^

N is the number of non-missing values. Percent (%) values follow the frequencies of the events n for HIV+ subjects with and without chronic kidney disease (CKD). P-value is for the differences between CKD and non-CKD groups. Tests used: ^1^Pearson test; ^2^Wilcoxon test. Abbreviations: CD4 = cell differential count; HIV-1 RNA = human immunodeficiency virus-1 ribonucleic acid; eGFR = estimated glomerular rate; OI/ADE = opportunistic infection/AIDs (autoimmune deficiency syndrome) defining event; IDU = intravenous venous use; ACEI/ARB = angiotensin converting enzyme inhibitor/angiotensin receptor blocker; highly active antiretroviral therapy (HAART). ^a^The median age at the first valid creatinine measurement. ^b^eGFR estimated glomerular filtration rate calculated using the CKD-EPI equation.^ c^ESRD diagnoses at baseline defined by ICD-9 coding (Refer to Methods). ^d^absolute CD4 count reported as cells/mm^3^. ^e^HIV-1 RNA reported as copies/ml. ^f^Comorbid conditions at baseline defined by ICD-9 Coding.

**Table 2.** Univariate and multivariate analyses for AIDS defining events (ADE) and death outcomes among HIV+ comprehensive care center (CCC) individuals based on chronic kidney disease (CKD) exposure, 1998 – 2005.

Baseline group (n)	Outcome*	Events (n)	Unadjusted incidence rate ratio (IRR) CKD: No CKD (95% CIs)	Adjusted hazard ratio (HR) CKD: No CKD**
Total population (2,127)	ADE and death	277	2.2 (1.3, 3.6)	1.3 (0.5, 3.2)
	ADE	227	1.9 (1.0, 3.5)	1.0 (0.4, 3.1)
	Death	80	3.6 (1.7, 7.8)	1.6 (0.4, 7.3)

*Total population adjusted for the following baseline covariates: age, absolute CD4 count, Percentage CD4, HIV-1 RNA, race, gender, bmi at baseline, hypertension, anemia, HAART use, Hepatitis C, cardiovascular disease, ACEI/ARB use, diabetes, IDU HIV risk, and albumin at baseline. ^**^95% Confidence Intervals (CIs) reported for the corresponding HR using Cox regression.

**Table 3.** Time varying cox analyses for AIDS defining events (ADE) and death outcomes among HIV+ comprehensive care center (CCC) individuals based on chronic kidney disease (CKD) exposure, 1998 – 2005.

Baseline group (n)	Outcome*	Hazard value (95% CIs**)	p value
Total population (2,127)	ADE and death	0.9 (0.4, 1.9)	0.7
	ADE	0.9 (0.4, 2.1)	0.8
	Death	1.4 (0.4, 4.4)	0.6

*Total population adjusted for the following baseline covariates: age, absolute CD4 count, percentage CD4, HIV-1 RNA, race, gender, bmi at baseline, hypertension, anemia, HAART use, Hepatitis C, cardiovascular disease, ACEI/ARB use, diabetes, IDU HIV risk, and albumin at baseline. Subjects with no CKD at baseline were defined as having CKD when their eGFR was less than 60 ml/min/1.73 m^2^ and also declined to ≥ 50% compared with baseline eGFR. **95% Confidence Intervals (CIs) reported for the corresponding HR using time varying cox regression.

**Supplemental Table 2.** ADE diagnosis stratified by type of ADE.

ADE Diagnosis	eGFR ≥ 60 (n = 216)	eGFR < 60 (n = 11)	Combined p = 0.73 (n = 227)
Non-Hodgkins lymphoma (NOS)	0% (0)	0% (0)	0% (0)
Abnormal loss of weight	0% (1)	0% (0)	0% (1)
AIDS with dementia NOS	13% (29)	27% (3)	14% (32)
Bacterial pneumonia	0% (1)	0% (0)	0% (1)
Bacterial pneumonia unspecified	0% (1)	0% (0)	0% (1)
Bacterial pneumonia NOS	0% (1)	0% (0)	0% (1)
Burkitt’s lymphoma NOS	0% (0)	0% (0)	0% (0)
Burkitt’s lymphoma or tumor	1% (3)	0% (0)	1% (3)
Candidial esophagitis	14% (31)	0% (0)	14% (31)
Candidiasis of the esophagus	0% (1)	0% (0)	0% (1)
Carcinoma in situ of cervix uteri	0% (1)	0% (0)	0% (1)
CMV esophagitis	0% (1)	0% (0)	0% (1)
CMV retinitis	1% (2)	0% (0)	1% (2)
Cryptococcal meningitis	0% (1)	9% (1)	1% (2)
Cryptococcus disseminated	2% (5)	0% (0)	2% (5)
Cryptosporidiosis	0% (1)	9% (1)	1% (2)
Dementia	1% (3)	0% (0)	1% (3)
Dementia NOS	0% (1)	9% (1)	1% (2)
Excessive body weight loss	1% (2)	9% (1)	1% (3)
Herpes simplex esophagitis	1% (2)	0% (0)	1% (2)
Histoplasmosis	2% (5)	0% (0)	2% (5)
Histoplasmosis NOS	0% (1)	0% (0)	0% (1)
HIV encephalopathy	2% (4)	9% (1)	2% (5)
Cryptosporidium	0% (1)	0% (1)	0% (1)
Mycobacterium tuberculosis	0% (1)	0% (0)	0% (1)
Kaposi’s sarcoma	2% (4)	0% (0)	2% (4)
Kaposi’s sarcoma oral	1% (2)	0% (0)	1% (2)
Kaposi’s sarcoma skin	1% (2)	0% (0)	1% (2)
Lymphoma NOS	0% (1)	0% (0)	0% (1)
Lymphoma Stage I	0% (1)	0% (0)	0% (1)
Lymphoma Stage IV	0% (1)	0% (0)	0% (1)
MAC	3% (6)	0% (0)	3% (6)
MAC disseminated	0% (1)	0% (0)	0% (1)
Large cell lymphoma (malignant)	0% (1)	0% (0)	0% (1)
